# High incubation temperatures enhance mitochondrial energy metabolism in reptile embryos

**DOI:** 10.1038/srep08861

**Published:** 2015-03-09

**Authors:** Bao-Jun Sun, Teng Li, Jing Gao, Liang Ma, Wei-Guo Du

**Affiliations:** 1 Key Laboratory of Animal Ecology and Conservational Biology, Institute of Zoology, Chinese Academy of Sciences, Beijing 100101, People's Republic of China; 2 University of Chinese Academy of Sciences, Beijing, People's Republic of China

## Abstract

Developmental rate increases exponentially with increasing temperature in ectothermic animals, but the biochemical basis underlying this thermal dependence is largely unexplored. We measured mitochondrial respiration and metabolic enzyme activities of turtle embryos (*Pelodiscus sinensis*) incubated at different temperatures to identify the metabolic basis of the rapid development occurring at high temperatures in reptile embryos. Developmental rate increased with increasing incubation temperatures in the embryos of *P. sinensis*. Correspondingly, in addition to the thermal dependence of mitochondrial respiration and metabolic enzyme activities, high-temperature incubation further enhanced mitochondrial respiration and COX activities in the embryos. This suggests that embryos may adjust mitochondrial respiration and metabolic enzyme activities in response to developmental temperature to achieve high developmental rates at high temperatures. Our study highlights the importance of biochemical investigations in understanding the proximate mechanisms by which temperature affects embryonic development.

Temperature has pervasive effects on almost every aspect of life. Perhaps one of the most well-known effects is its influence on biological rates such as the rates of metabolism, development, and growth^1^. Despite the overwhelming number of studies describing the thermal dependence of these biological rates^1^,^2^,^3^, the underlying mechanisms driving the relationship between temperature and the rates are still elusive. For example, the temperature-size rule predicts that growth efficiency decreases as environmental temperature increased^4^, but empirical studies show that growth efficiency is either positively related or insensitive to environmental temperature in most ectotherms^5^.As the centre of an organism's energy supply, mitochondria are critical for understanding the biochemical and physiological processes underlying temperature effects on the biological rates^1^,^6^. A number of studies have demonstrated that temperature may affect mitochondrial properties and the activities of metabolic enzymes in different lineages of animals (e.g. insects^7^; fish^8^; frogs^9^,^10^). The majority of these studies focus on the effect of temperature on physiological and biochemical properties of post-hatching individuals. For example, developmental temperature modulate thermal acclimation of performance curves of locomotion as well as metabolic enzymes [e.g. lactate dehydrogenase (LDH), citrate synthase (CS) and cytochrome *c* oxidase (COX)] in tadpoles of the frog, *Limnodynastes peronii*^9^. However, little is known how developmental temperature affects the biochemical responses of embryos such as the activity of metabolic enzymes.

The development of reptilian embryos involves two distinct but related processes: differentiation and growth^11^. The developmental rate of embryos is enhanced with increasing temperature, and this temperature effect is universally observed in almost all studied species of ectothermic animals such as insects, fish, and reptiles^3^,^12^. As in other ectothermic animals, developmental rate increases exponentially with temperature in reptile embryos^13^. For example, developmental rate is enhanced with increasing temperature in the embryos of the Chinese softshell turtle *Pelodiscus sinensis*, a species with a widespread distribution across China and Southeast Asia (Fig. 1a). In addition, the instantaneous developmental rate (i.e., the slope of the developmental curve) increases with temperature, and the among-temperature difference in instantaneous developmental rate becomes larger as embryonic development proceeds over most of the incubation period (70%) in this species (Fig. 1b). How might turtle embryos develop more rapidly at high temperatures? The developmental rate depends on the metabolic rate, that is, the rates of energy utilization, transformation, and allocation^14^,^15^, which are in turn related to the function of mitochondria^16^.To enhance its developmental rate, an embryo may increase energy metabolism with increasing temperature. In addition to this same general rule of biochemical reactions underpinning all life activities, we envisage another potential pathway that an embryo might use to enhance its developmental rate. That is, the mitochondrial energy metabolism of embryos at high temperatures could be further enhanced through developmental plasticity or thermal acclimation, because both developmental plasticity^17^,^18^ and thermal acclimation^9^ may modify physiological and biochemical responses to temperature. In this study, we incubated *P. sinensis* eggs under 24, 28 and 32°C respectively and measured mitochondrial respiration, activities of aerobic metabolic enzyme (COX) and anaerobic metabolic enzyme (LDH) of *P. sinensis* embryos at different test temperatures of 24, 28 and 32°C. By determining the effects of incubation and test temperatures on mitochondrial traits and metabolic enzyme activities, we aimed to identify how turtle embryos enhance developmental rate at high temperatures, and thereby test for the proposed hypothesis.

## Results

### Mitochondrial respiration

Both State 3 (*F*_2, 30_ = 68.11, *P* < 0.0001) and State 4 (*F*_2, 30_ = 60.13, *P* < 0.0001) respirations were enhanced as test temperature increased. Incubation temperature also significantly affected mitochondrial respiration, with embryos incubated at high temperature showing greater State 3 (*F*_2, 15_ = 37.25, *P* < 0.001) and State 4 (*F*_2, 15_ = 31.78, *P* < 0.001) respirations than those incubated at low temperature (Fig. 2a,b).

### Metabolic enzyme activity

The COX activity of embryos was enhanced in response to the increase in test temperature (*F*_2, 30_ = 246.43, *P* < 0.0001) as well as incubation temperature (*F*_2, 15_ = 101.52, *P* < 0.0001), and was more strongly enhanced for embryos from the high incubation temperature group compared to those from the low incubation temperature group (Fig. 2c). Similarly, LDH activity was enhanced as test temperature increased (*F*_2, 30_ = 106.25, *P* < 0.0001), and was higher in embryos incubated at 28°C and 32°C than those incubated at 24°C (*F*_2, 15_ = 10.308, *P* = 0.002) (Fig. 2d).

## Discussion

**Figure 1:**
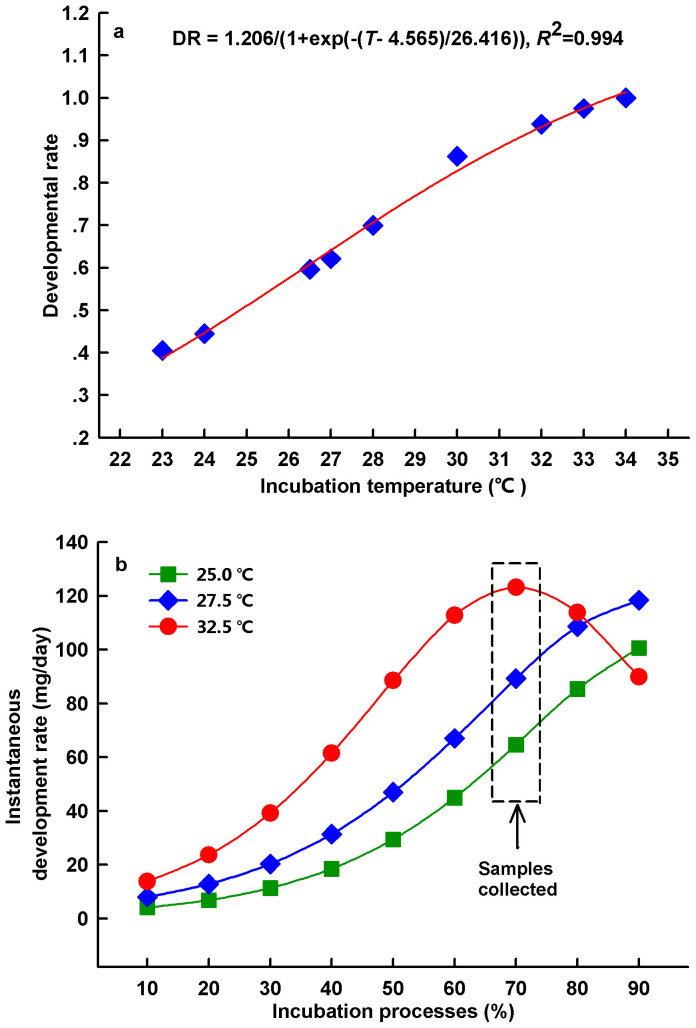
Developmental rates (a) and instantaneous developmental rates (wet mass) (b) of embryos incubated under different temperatures. Developmental rate (DR) at each temperature (T) was calculated by dividing the incubation duration by the shortest incubation duration recorded in the laboratory, and taking the inverse of this value. Data were obtained from Du and Ji, 2003 (a)^35^ and from Yang et al., 2002 (b)^36^.

**Figure 2:**
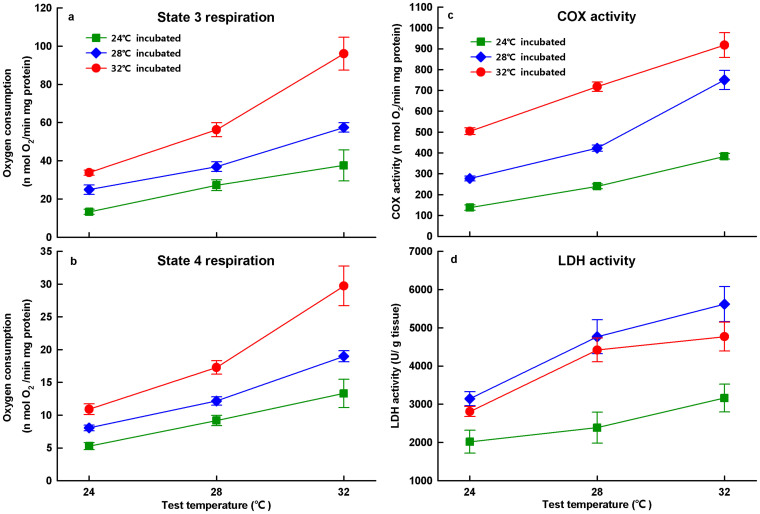
Mitochondrial respirations [State 3 (a) and 4(b)] and enzyme activities [cytochrome *c* oxidase, COX (c) and lactic dehydrogenase, LDH (d)] of embryos incubated at 24°C (square), 28°C (diamond), and 32°C (circle), respectively. Mitochondrial functions and enzyme activities increased as test temperatures increased, and showed thermal plasticity in response to incubation temperature. N = 6 for each incubation temperature, and the significance level was defined as *P* < 0.05.

Consistent with our predictions, mitochondrial respiration and COX activities in *P. sinensis* embryos were enhanced not only by acute temperature increases but also by exposure to high incubation temperatures during development, demonstrating the thermal dependence of developmental rate (Fig. 1 & Fig. 2). Our results suggest that developmental temperature may adjust mitochondrial respiration and enzyme activities, with better mitochondrial performance observed at higher incubation temperatures in reptile embryos. In future studies, exploring the functional mechanisms underlying the thermal plasticity of embryonic mitochondrial traits would be of great interest.

The developmental thermal plasticity of physiological and biochemical traits in embryos differs from the process of thermal acclimation of these same traits in post-embryonic individuals. High developmental temperature enhanced mitochondrial respiration and enzyme activities (Fig. 2). This may explain why the instantaneous developmental rate increases more rapidly at high temperatures than at low temperatures as embryonic development progresses. Specifically, increased mitochondrial respiration and aerobic enzyme activities may accelerate mitochondrial oxygen consumption and catalytic activity, and then improve aerobic metabolism to produce more energy resources (ATP) in the mitochondria^16^,^19^. By contrast, at the post-embryonic stage, individuals show an opposite pattern with respect to thermal acclimation of metabolism and enzyme activities. In general, respiration rate and enzyme activity are higher in cold-adapted or cold-acclimated individuals than in warm-adapted or warm-acclimated individuals, although this is not universally true^6^. For example, the activities of LDH and citrate synthase (CS) are two- to three-fold higher in Antarctic fish than in tropical fish^1^. Thermal acclimations of COX, CS, and LDH activities in *Alligator mississippiensis* serve to maintain physiological performance, even at unfavourably cold temperatures^20^. This kind of discrepancy between life-history stages may also exist in other physiological traits such as heart rate, which changes ontogenetically, with a higher increasing rate observed in embryos than hatchlings^21^. From an evolutionary perspective, this discrepancy may reflect divergent patterns of energetic allocation between embryonic and post-embryonic development, which are likely subjected to different selective forces; embryos have access to sufficient energy storage (yolk), whereas post-embryonic individuals do not^21^.

In nature, most turtles dig their nests at warm open sites^22^,^23^. Achieving high developmental rates at high temperatures may thus have important ecological consequences. First, high developmental rate likely increases egg survival, because shorter incubation periods reduce the risk of an embryo exposed to environmental hazards (e.g. extreme temperature, dry condition, and predation)^24^. Second, high developmental rate means earlier hatching, which will likely enhance offspring viability in many reptiles. For example, early-emerging offspring have access to more food, face less competition, have longer time to grow before winter, and therefore increase the survival rate of hatchlings^25^,^26^,^27^,^28^.

Our study demonstrated that turtle embryos may adjust physiological and biochemical characteristics such as mitochondrial respiration and enzyme activities to enhance their developmental rate, thus highlighting the importance of biochemical investigations in understanding the proximate mechanisms by which temperature affects embryonic development. Reptile embryos can develop across a wide range of temperatures from approximately 10°C to 45°C^29^, and thus provide ample opportunity as a model organism to deepen our understanding of thermal adaptation at the biochemical and molecular levels in addition to investigating the effects of temperature on embryonic development at the individual level in oviparous reptiles (see review by Deeming^13^).

## Methods

### Ethic statements

This research was performed in accordance with the NIH *Guide for the Principles of Animal Care*. The protocol and study were approved by the Animal Ethics Committee at the Institute of Zoology, Chinese Academy of Sciences (Permit Number: IOZ14001).

### Egg collection and incubation

Freshly laid *Pelodiscus sinensis* eggs were collected from a private hatchery in Baoding (Hebei province, North China). Fertilized eggs (with a white patch; n = 180) were weighed and incubated in incubators (KB240; Binder, Germany) maintained at three constant temperatures of 24°C, 28°C, and 32°C, which correspond to the low, medium, and high temperatures experienced by eggs in field nests, respectively. Eggs were assigned to the three thermal treatments using a split-clutch design, and were incubated in 160 × 105 × 45 mm(length × width × height) plastic boxes with moist vermiculite(−220 kPa; water:vermiculite = 1.1:1 in mass). Water was added to the vermiculite every four days to compensate for evaporative losses. Toward the end of the incubation period, we checked the boxes every day for newly emerging hatchlings. The incubation period was calculated as the number of days elapsed between the beginning of incubation and the emergence of the hatchlings.

### Tissue collection, homogenate and isolation of mitochondria

When incubation was approximately 70% complete, 20 eggs from each incubation temperature were dissected for tissue collection. The livers of embryos were then collected and weighed (±0.1 mg). For isolation of mitochondria, two or three fresh livers from different embryos in the same thermal treatments were pooled together as one sample, and the sample size of mitochondrial suspension in each incubation temperature was equally six. We isolated the mitochondria using established methods with some modifications^30^,^31^. In brief, the livers were immediately homogenized with a homogenizer (T10;IKA, Germany) in ice-cold extraction buffer (250 mM sucrose, 5 mM Tris/HCl, and 2 mM EGTA, pH 7.4, at 4°C). The homogenate was centrifuged at 1000 × g for 10 min at 4°C, and the precipitate was discarded. The residual supernatant was centrifuged at 12,000 × g for 10 min at 4°C, and then the precipitate was resuspended with the extraction buffer. The high-speed centrifugal cycle was then repeated twice. Finally, the mitochondrial extraction was obtained by resuspending the pellet of isolation buffer (120 mM KCL, 3 mM HEPES, 5 mM KH_2_PHO_4_ and 1 mM EGTA, pH 7.2, at 4°C) in a volume of 0.3 ml.

### Mitochondrial respiration

Mitochondrial respiration in liver mitochondria was measured polarographically with a temperature-controlled Clark electrode system (Hansatech Instruments; UK). Each mitochondrial suspension of liver from embryos incubated under different temperatures were assayed for mitochondrial respiration at 24°C, 28°C, and 32°C respectively, according to established methods^30^,^32^ with minor modifications. The mitochondrial respiration system comprised a 1 ml solution (0.94 ml medium and 0.06 ml mitochondrial suspension) for State4 and State3 respiration measurements. After the solution was established, 1 μl rotenone (5 μM) was added into the system to restrict the respirations of complex I, so the endogenous substrate reaction was terminated^16^. The state 4 respiration was substrate-dependent, and 4 μl succinate (4 mM) was used as a substrate. Then, 2 μl ADP (200 μM) was added to establish the State3 respiration^30^,^32^,^33^.

### Metabolic enzyme activity assay

The activities of COX and LDH were measured in duplicate at test temperatures of 24°C, 28°C, and 32°C. COX activity of the mitochondrial extraction was also determined polargraphically. COX activity was measured according to published protocols^30^,^34^. In brief, the reactive system comprised 1.98 ml of medium and 0.01 ml mitochondrial suspension. Then 0.01 ml cytochrome C was added to establish reaction. For anaerobic metabolic enzyme (LDH) assay, we homogenized fresh livers of six embryos from each incubation temperature with buffer on ice, according to the specification of LDH assay kit (Huili; Changchun, China). LDH activity of liver homogenate was measured using the assay kit with an ultraviolet/visible spectrophotometer (Jinghua 75;Shanghai, China). LDH activities were calculated on the basis of the linear portion of the reaction rates at 510 nm and expressed as U/g tissue.

### Statistical analysis

The thermal dependence of mitochondrial respirations (State 3 and State 4), COX and LDH activities for embryos incubated at different temperatures was analysed using repeated-measures ANOVAs, with test temperature as the repeated measure and incubation temperature as the main factor. Further *post-hoc* Tukey HSD tests were carried out to determine the among-treatment differences.

## Author Contributions

B.-J.S. and W.-G.D. designed the study, analyzed the data and wrote the manuscript. B.-J.S., T.L., J.G. and L.M. performed the experiments.
